# Comparison of workloads in two nursing care delivery models in adult intensive care units

**DOI:** 10.15649/cuidarte.4510

**Published:** 2025-12-18

**Authors:** Katherine Rojas Cárdenas, William Iván López Cárdenas, Natalia Andrea Henao Murillo

**Affiliations:** 1 Egresada, Universidad de Antioquia, Medellín, Colombia. E-mail: katherine.rojasc@udea.edu.co Universidad de Antioquia Medellín Colombia katherine.rojasc@udea.edu.co; 2 Docente, Universidad de Antioquia, Medellín, Colombia. E-mail: wivan.lopez@udea.edu.co Universidad de Antioquia Medellín Colombia wivan.lopez@udea.edu.co; 3 Docente, Universidad de Antioquia, Medellín, Colombia. E-mail: nathyh75@gmail.com Universidad de Antioquia Medellín Colombia nathyh75@gmail.com

**Keywords:** Critical Care Nursing, Hospital Nursing Staff, Nurses, Nursing Assistants, Workload, Enfermería de Cuidados Críticos, Personal de Enfermería en Hospital, Enfermeras, Asistentes de Enfermería, Carga de Trabajo, Enfermagem de Cuidados Críticos, Pessoal de Enfermagem em Hospital, Enfermeiras, Assistentes de Enfermagem, Carga de Trabalho

## Abstract

**Introduction:**

Work overload is associated with missed care and a higher prevalence of adverse events. The study aims to generate empirical evidence on nursing workload in intensive care units in Colombia, considering differences in team composition based on qualifications, skills, and competencies (Skill Mix).

**Objective:**

To compare the workload and distribution of nursing activities in two intensives cares units with different Nursing Care Models based on the application of the TISS-28.

**Materials and Methods:**

Quantitative and retrospective study with data from medical records of patients treated between 2018 and 2020 in Medellín. The TISS-28 was calculated upon admission and SAPS-3 was used to control for confounding variables. Mean and proportion comparison tests were used according to the type of variable and data distribution pattern.

**Results:**

The nurses in both ICUs had a patient assignment higher than indicated by the TISS-28. Surgical patients, those with vasoactive drugs, transfusions, adverse events, or those who died, had longer care times. The adverse events increased proportionally with the TISS-28 level.

**Discussion:**

Key elements include the relationship between care safety and staff qualification, experience and workload; the missed nursing care; and the undervaluation of care as a public good.

**Conclusion:**

It was identified that in both ICUs there is a higher workload for nurses regarding the time available for activities and patients. The majority of care provided by the nursing assistants in the ICU2-Assistants do not correspond with their level of training and legal responsibility.

## Resumen

**Introducción:**

La sobrecarga de trabajo se asocia con la omisión del cuidado y mayor prevalencia de Eventos Adversos. El estudio pretende generar evidencia empírica sobre la carga de enfermería en unidades de cuidado intensivo en Colombia con diferencias en la composición del equipo por cualificaciones, habilidades y competencias (Skill Mix).

**Objetivo:**

Comparar la carga de trabajo y la distribución de actividades de enfermería en dos UCI con diferentes Modelos de Atención de Enfermería basados en la aplicación del TISS-28.

**Materiales y métodos:**

Estudio cuantitativo y retrospectivo con datos de atenciones clínicas entre 2018 y 2020 en Medellín. Se calculó el TISS-28 al ingreso y se usó Simplified Acute Physiology Score 3 (SAPS-3) para controlar variables confusoras. Se utilizaron pruebas de comparación de medias y proporciones según el tipo de variable y patrón de distribución de los datos.

**Resultados:**

La asignación de pacientes en ambas UCI fue superior a la indicada por el TISS-28. Los pacientes quirúrgicos, con vasoactivos, transfusiones, eventos adversos (EA) o que murieron requirieron mayores tiempos de cuidado. Los EA aumentaron de forma proporcional con el nivel del TISS-28.

**Discusión:**

Destacan elementos como la relación entre la seguridad del cuidado y el nivel de formación, experiencia del personal y carga de trabajo; la omisión de cuidados y la subvaloración del cuidado como bien público.

**Conclusiones:**

Se identificó que en ambas UCI hay más carga de trabajo para las enfermeras respecto al tiempo disponible por actividades y pacientes. La mayor parte de cuidados brindados por las auxiliares de enfermería de la UCI2-Auxiliares no corresponden con su nivel de formación y de responsabilidad legal.

## Resumo

**Introdução:**

A sobrecarga de trabalho está associada à negligência dos cuidados e à maior prevalência de eventos adversos. Este estudo tem como objetivo gerar evidências empíricas sobre a carga de trabalho de enfermagem em unidades de terapia intensiva na Colômbia, com diferenças na composição da equipe por qualificações, habilidades e competências (Skill Mix).

**Objetivo:**

Comparar a carga de trabalho e a distribuição das atividades de enfermagem em duas UTIs com diferentes Modelos de Assistência de Enfermagem, a partir da aplicação do TISS-28.

**Materiais e métodos:**

Estudo quantitativo e retrospectivo com dados de atendimentos clínicos entre 2018 e 2020 em Medellín. O TISS-28 foi calculado na admissão, e o Simplified Acute Physiology Score 3 (SAPS-3) foi utilizado para controlar variáveis de confusão. Testes de comparação de médias e proporções foram utilizados de acordo com o tipo de variável e o padrão de distribuição dos dados.

**Resultados:**

A alocação de pacientes em ambas as UTIs foram maiores do que a indicada pelo TISS-28. Pacientes submetidos a cirurgias, agentes vasoativos, transfusões, eventos adversos (EA) ou óbitos necessitaram de maior tempo de cuidado. Os EA aumentaram proporcionalmente com a pontuação do TISS-28.

**Discussão:**

Os elementos destacados incluem a relação entre a segurança do cuidado e o nível de treinamento, experiência da equipe e carga de trabalho; a omissão de cuidado; e a subvalorização do cuidado como um bem público.

**Conclusões:**

Foi identificado que, em ambas as UTIs, os enfermeiros têm maior carga de trabalho em comparação ao tempo disponível para atividades e pacientes. A maioria dos cuidados prestados pelos auxiliares de enfermagem na UTI2-Auxiliares não corresponde ao seu nível de treinamento e responsabilidade legal.

## Introduction

The quality of care and the clinical outcomes of patients are related to the ways in which nursing staff organize their work - represented in Nursing Care Models (NCMs) - however, there is little clarity on the ideal allocation of workload for such staff, especially because it is affected by different attributes such as the amount of nursing time and functions assigned, the level of knowledge and skills, the complexity of care, the weight of the intensity of care and the physical, mental and emotional effort^1^-^3^.

Workload analysis allows nursing managers to calculate staffing and patient allocation in the Intensive Care Unit (ICU) considering the needs of each patient and the severity of their health condition^4^-^5^. Scientific literature shows that assigning nursing teams to patient care without determining the workload with validated instruments is correlated with decreased quality of care and patient safety, expressed in indicators such as adverse events (AEs) in the ICU, and omission of care^6^-^7^ .

Research conducted in Egypt, Western Australia, South Korea, and Brazil showed a positive correlation between workload and neglect of care, due to the high number of patients per nurse, poor infrastructure, and staff with multiple employment contracts^6^-^9^. In contrast, research focused on identifying the association between the use of nursing aids and nurse-patient outcomes showed that implementing a mandatory minimum nurse-to-patient ratio^8^ was associated with or correlated with improvements in indicators such as job satisfaction and a reduction in adverse events such as healthcare-associated infections, pressure ulcers, and patient falls^10^.

In Colombia, the study by Arango was found^11^, whose correlation analyses were inconclusive in establishing a correlation between patient allocation and some indicators of quality of healthcare. A group of studies with design limitations was also identified, such as setting comparative objectives but presenting unified results without disaggregating any group^12^, proposing descriptive approaches without establishing relationships between variables^13^, applying statistical tests that do not correspond to the data distribution pattern, the quantitative-qualitative nature of the information, or the data tabulation method (e.g., applying Student's t -tests to compare three data groups)^5^.

Regarding the nurse-patient ratio, territories such as the United States, the United Kingdom, and Australia have established 1:1 standards for ICUs^14^ ; however, the regulations of the Colombian health system have not established a nurse-patient ratio, and therefore, hospitals consider hiring nursing technicians for the direct care of patients under the supervision of a nurse per ward, who has legal responsibility for patient care and also performs administrative functions^1^,^15^. Adding to this situation is the limited availability of nurses and inequities in their geographical distribution, as they are mostly concentrated in large urban centers^16^.

In Colombian ICUs, there is a greater availability of nurses for direct care compared to inpatient services due to technological complexity and patient care requirements. However, workload overload has been observed related to the limited availability of nurses, the number of duties, and the severity of the patients assigned^17^. A heterogeneous range of nurse-patient assignments has been found, ranging from 1-3, allowing nurses to participate in direct patient care, to 1-12, where the nurse is limited to ward management and loses the central role in patient care, delegating all care to the nursing assistants under their supervision^18^. Regarding the distribution of tasks, G. Arango^11^ describes that nurses perform few direct care activities, mainly medication administration and transfusion supervision. The rest of their time is spent on administrative tasks, and most of the care is provided by nursing assistants without the technical and legal competence for the functions they perform.

Given the lack of regulations on the nurse-to-patient ratio, the use of validated instruments such as the Therapeutic Intervention Scoring The system (TISS-28) allows nursing managers to calculate workloads and assign nursing teams according to the severity level and care requirements of patients^13^,^19^. One aspect to highlight is that the different studies reviewed in Colombia agree in showing the work overload on nursing staff, although none of them provides a description of the workload in relation to the composition of the work teams by levels of qualifications, skills and competencies (Skill Mix) and the distribution of activities of TISS-28^5^,^11^,^12^,^20^.

Considering the methodological limitations of some of the studies carried out in the Colombian context, the heterogeneity in the nurse-patient ratio and the diffuse and unregulated boundaries on the functions of nurses and nursing assistants in hospital care, the development of local knowledge is required that integrates these factors in a relational way and generates knowledge for action, that is, studies with epistemic vigilance on the methodological rigor itself and that study the workload in ideal scenarios regarding the allocation of patients and functions between nurses and nursing assistants.

Given the need to establish nurse-patient assignments that align with care needs, some Colombian hospitals have made progress in implementing multi-patient assignment models (NCMs) with a higher proportion of registered nurses, fewer assigned patients, and a distinction in the roles of nurses and nursing assistants based on their qualifications and legal responsibilities: nurses for direct care and auxiliary staff for comfort support activities. Analyses of workloads in these new NCMs are limited, making them an emerging field for research on the relationship between workload, roles, and quality of nursing care^19^.

This led to an interest in conducting a comparative study in two Intensive Care Units (ICUs) of a high- complexity hospital in Medellín, Colombia, with different organizational structures for their nursing teams. In one ICU, nurses provide direct care with support from nursing technicians for comfort care activities, while in the second ICU, a nurse is responsible for the ward and supervises the nursing technicians, who provide most of the patient care, including tasks for which they lack the necessary skills, such as medication administration, infusion preparation, and some invasive procedures. The units differ in the proportion of nurses and nursing assistants per team, the number of patients, and their assigned roles. To identify similarities or differences in workload under these organizational structures of nursing care, the following study objective was established: to compare the workload and distribution of nursing activities in two ICUs with different Nursing Care Models based on the application of the TISS-28.

## Materials and Methods

**The design and location of the study**

This quantitative, descriptive, and retrospective study used data from the medical records of patients in two ICUs at a hospital in Medellín between December 2018 and March 2020, a period corresponding to the institutional piloting of two nursing care models for adult ICUs. The ICUs differed in the composition, skills, and functions within their nursing teams and were designated ICU1-Nurses and ICU2-Nursing Assistants, based on the profile that was more numerous and performed the greatest proportion of direct patient care in each ICU.

**Population**

One thousand Electronic Health Records (EHRs) of patients over 18 years of age, with a hospital stay longer than 48 hours and with a single admission to the ICU during their hospital stay, were included. For the sample size calculation, the average number of adverse events (AEs) in each ICU between January 2018 and August 2019 was used, with a standard deviation (SD), a power of 90%, a type of alpha error of 5%, and an adjustment for losses of 20%, determining a minimum of 348 admissions in each group.

**Variables, instruments, and data collection procedures**

A data collection protocol was in place for reviewing medical records, applying instruments, and training the data collection personnel. Data collection took place between July 2021 and June 2023. Sociodemographic variables of the patients were collected, as well as adverse events (AEs) experienced during their ICU stay. The probability of death, and the duration and care requirements based on the severity of the patient's condition during the first day of ICU stay, were calculated using the Simplified Acute Physiology Score (SAPS-3). Therapeutic Intervention Score System TISS-28, both validated for the Colombian and South American context^20^.

The TISS-28 has six domains: basic activities, ventilatory support, cardiovascular support, renal support, neurological and metabolic support, and specific interventions^21^,^22^. The total score can range from 0 to 76 points and classifies patients into four severity levels, each with a specific nurse-to-patient ratio: Class I with a 1:4 ratio; Class II 1:4; Class III 1:2; and Class IV with a 1:1 or 2:1 nurse-to-patient ratio. Classes I and II require observation or active monitoring, while Classes III and IV require intensive care and therapy^21^.

**Data analysis**

For qualitative variables, absolute and relative frequencies were calculated, and for quantitative variables, measures of central tendency and dispersion were calculated. Once normality in the distribution of the variables was determined, chi-square tests and corresponding mean comparison tests were performed. The data included both within-group and between-group comparisons. All collected data are freely available for access and consultation on Mendeley Data^23^.

**Validity criteria**

To ensure internal validity, a protocol was developed using validated instruments, and the data collection staff was trained and supervised. Weekly audits were conducted, and the quality of 20% of the medical records collected was analyzed. The SAPS-3 was used to ensure patient comparability and control for confounding biases related to demographic and clinical characteristics.

**Ethical considerations**

This research was deemed safe according to Colombian Resolution 8430/1993. Approval was obtained from an Ethics Committee (CEI-FE 2019 minutes), adhering to the legal confidentiality criteria of the HCL. The confidentiality of the hospital's name was guaranteed, as well as the anonymized use of sensitive patient information using alphanumeric identification codes and the removal of variables such as name and identification number.

## Results

**Characteristics of the nursing staff participating in ICUs**

The main differentiating characteristic of the working methods in the ICUs compared is skill staffing mix (proportion of professional staff on teams and assignment of roles). The proportion of nurses in ICU 1-Nurses is three times greater than in ICU 2-Nursing Assistants. In ICU 1-Nurses, one nurse is responsible for three patients and one nursing assistant for four patients. There are four nurses and three nursing assistants per shift to cover the unit's 12 beds, in addition to an administrative coordinator on day shifts and weekdays, who oversees the service's administrative management (Table 1). In ICU 2-Nursing Assistants, there is one nurse per shift, responsible for the care of 13 patients, with six nursing assistants, most of whom care for two patients (Table 1).

The distribution described places the nurses in ICU1-Nurses in a direct care role with support from nursing assistants for comfort activities, while in ICU2-Auxiliaries the nurse has a management- administration profile of the ward and delegates most of the care to the nursing assistants, except for taking blood cultures and assisting in invasive procedures.

**Table 1:** Characterization of nursing staff and ICUs compared

ICU variables	ICU 1 Nurses	ICU 2 Assistants
Nursing staff allocation per patient		
Nurses	1:3	1:13
Assistants	1:4	1:2
Available beds	12	13
Number of staff per shift		
Nurses	4	1
Assistants	3	6
Coordinator	1	0
Rotating bed	7.44 days / patient	9.24 days / patient
**Nursing variables**	**ICU 1 Nurses**	**ICU 2 Assistants**
	n Median (IQR)	n Median (IQR) p-value
Age (years)		
Assistants	7 23 (4)	17 33 (7) <0.001*
Nurses	17 34 (13)	5 30 (8) 0.249*
Years of experience		
Assistants	7 3 (0.0)	17 5 (7.5) 0.494*
Nurses	17 3 (10.5)	5 1 (1.5) 0.002*
Skill Mix n [%]		0.005~
Assistant	7 [29.20]	17 [77.30]
Nurse	9 [37.50]	3 [13.60]
Specialist	8 [33.30]	2 [9.10]

ICU-Intensive Care Unit, IQR; Interquartile range, ~ p value for chi square * p value for Mann's U Whitney.

The sociodemographic characteristics of the work teams show that in ICU 1-Nurses, the nurses are older and have similar professional experience compared to the nursing assistants (Table 1), while in ICU 2-Assistants, the auxiliary staff are older and have more experience. The dispersion measures for years of experience suggest that within the teams, there is a coexistence of highly experienced individuals and a constant turnover of new staff with little work experience. The bed turnover rate indicates longer hospital stays in ICU 2-Assistants.

**Patient characteristics and workload according to TISS-28**

In the two ICUs compared, a similar mean age was found, along with a higher proportion of men and discharges. Patients did not show significant differences in their demographic and clinical characteristics, except for the proportion of scheduled admissions, surgical and cardiovascular patients, and the time spent on the TISS-28, suggesting that patients in ICU 1-Nurses had greater care requirements. A direct proportional increase was also found between the TISS-28 level and the percentage of adverse events, which were significantly higher for ICU 2-Auxiliaries (Table 2).

**Table 2:** Characteristics of patients treated during the study period. n=1000

Variable	ICU 1 Nurses % (n) 541	ICU 2 Assistants % (n) 459	p-value
Sex			0.689*
Male	55.82 (302)	57.08 (262)	
Female	44.18 (239)	42.92 (197)	
Age (years) - Mean ± SD	52 ± 19.70	52.4 ± 19.10	0.856~
Type of income			<0.001*
Programmed	22.92 (124)	13.73 (63)	
Urgent	77.08 (417)	86.27 (396)	
Type of discharge			0.255*
High	85.95 (465)	82.14 (377)	
Death	14.05 (76)	17.86 (82)	
Probability of death			0.854~
SAPS 3 (%)	47.80 (NA)	48.10 (NA)	
Health regimen			0.44
Contributory	41.04 (222)	37.47 (172)	
Subsidized	53.60 (290)	55.99 (257)	
Not affiliated	5.36 (29)	6.54 (30)	
Place of origin			0.03
Emergency room, another hospital	59.10 (320)	65.80 (302)	
Another place in the hospital	40.85 (221)	34.20 (157)	
Days of hospitalization prior to admission to the ICU			0.18
0 to 13 days	93.16 (504)	95.86 (440)	
14 to 27 days	5.36 (29)	3.27 (15)	
Greater than or equal to 28 days	1.48 (8)	0.87 (4)	
Admitted for digestive pathology			0.86
No	89.54 (479)	88.89 (408)	
Yes	11.46 (62)	11.11 (51)	
Admitted for a liver condition.			0.94
No	97.97 (530)	98.04 (450)	
Yes	2.03 (11)	1.96 (9.00)	
Admitted for a neurological condition.			0.14
No	60.63 (328)	65.14 (299)	
Yes	39.37 (213)	34.86 (160)	
Admitted for surgery			<0.001
Programmed	22.92 (124)	15.47 (71)	
Urgent	29.57 (160)	21.35 (98)	
Non-surgical	47.50 (257)	63.18 (290)	
Admitted for cardiovascular pathology			0.01
No	78.19 (423)	70.37 (323)	
Yes	21.81 (118)	29.63 (136)	
TISS Average 28			0.003~
Mean Score ± SD	32 ± 9.10	30.2 ± 9.10	
TISS Category 28			0.011*
II = 10-19 points	9.43 (51)	13.51 (62)	
III = 20-39 points	67.28 (364)	69.72 (320)	
IV => 40 points	23.29 (126)	16.78 (77)	
Minutes of care required Mean ± SD	330 ± 94.30	309.00 ± 93.70	<0.001~
Adverse events by TISS-28 category			<0.001*
II	25.50 (13)	38.70 (24)	
III	56.90 (207)	66.90 (214)	
IV	85.70 (108)	92.20 (71)	

ICU-Intensive Care Unit, SD- Standard Deviation, SAPS 3- Simplified Acute Physiology Score, TISS 28- Simplified Therapeutic Intervention Score System, * p-value for chi square ~ p-value for Mann Whitney U.

Applying the TISS-28 to patient medical records resulted in a slightly higher overall average and care time in the ICU1-Nurses unit. Regarding severity level, approximately 70% of patients were classified as TISS-28 category 3. Patient assignments to nursing assistants in both ICUs corresponded to the ideal assignment calculated using the TISS-28, while for nurses, it revealed work overload; consequently, the number of patients assigned exceeded the ideal value based on the TISS-28 (Table 3).

**Table 3:** Care times, nursing staff-patient ratio and proportion of patients by TISS-28 categories

TISS-28 Category	Job profile	ICU 1 Nurses				ICU 2 Assistants			
		Median [IQR] minutes of care required	Staff-to-patient ratio		Patients treated % (n)	Median [IQR] minutes of care required	Staff-to-patient ratio		Patients treated % (n)
			Ideal TISS-28	Real			Ideal TISS-28	Real	
2	Nurse AN	101 [60-145] 54 [53-80]	1:5 1:9	1:3 1:4	9.40 (51)	42 [32-99] 108 [81-152]	1:11 1:4	1:13 1:2	13.50 (62)
3	Nurse AN	222 [116-318] 96 [53-113]	1:2 1:5	1:3 1:4	67.30 (364)	112 [47-190] 192 [112-281]	1:4 1:2	1:13 1:2	69.70 (320)
4	Nurse AN	337 [294-447] 108 [92-125]	1:1 1:4	1:3 1:4	23.30 (126)	184 [150-219] 258 [212-312]	1:3 1:2	1:13 1:2	16.80 (77)

ICU-Intensive Care Unit, IQR- interquartile range, TISS 28 - Simplified Therapeutic Intervention Score System, AN-Auxiliary nurse.

**Distribution of functions and care times according to TISS-28 activities**

**Figure 1:**
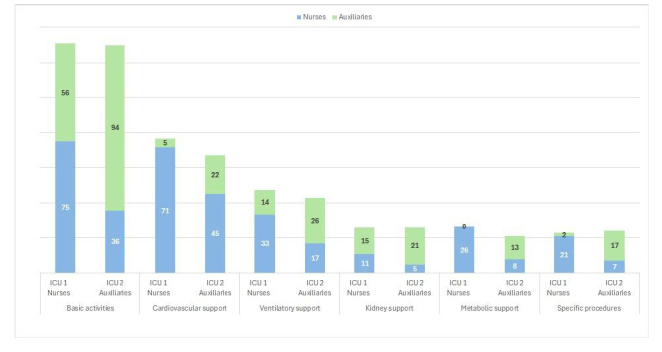
Time in minutes for general groups of TISS-28 activities

When analyzing care time across the six activity groups distributed between nurses and nursing assistants, it was observed that basic care consumed the most time, followed by cardiovascular and ventilatory support in both units. In ICU 1-Nurses, the nurse had the greatest involvement in all care activities, while in ICU 2-Assistants, it was the nursing assistants, except for cardiovascular support activities, where the nurse was more involved (Figure 1).

The analysis of specific activities of the TISS-28 shows that in the ICU1-Nurses, nurses perform activities such as the administration of medications and vasoactive drugs, ventilatory support, invasive procedures such as taking laboratory samples, nutritional support, the management of wound care, drains and catheters, CPR and assistance to intra-ICU procedures, while the nursing assistant has greater participation in activities such as patient monitoring, fluid balance and actions not contemplated in the TISS-28 such as assistance in basic activities of daily living.

In the case of the ICU2-Auxiliary Unit, the nurse performs invasive procedures such as the management of Catheters, complex wound care, sample collection, and assistance with intra - ICU medical procedures are performed by nursing assistants, while nursing assistants provide assistance with activities of daily living and additionally offer care such as medication administration, management of metabolic complications, nutritional support, ventilatory support, and artificial airway management. (Figure 1)

**Figure 2:**
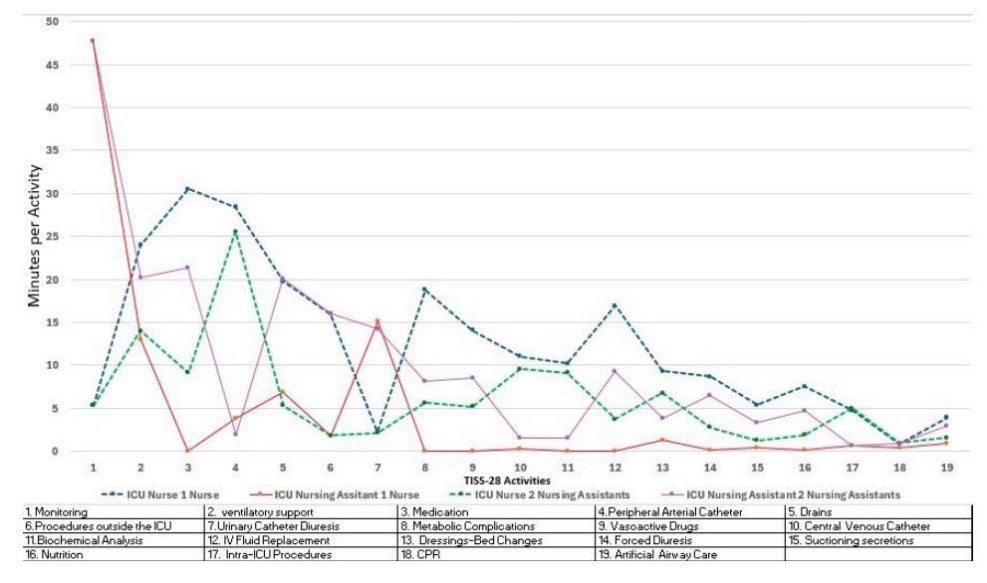
Time in minutes per activity

Figure 2 shows three trends regarding the care provided by nurses and nursing assistants in the two ICUs. The first trend reveals a similarity in the functions and roles of both nursing assistants and nurses in both ICUs, in activities such as bladder catheterization, management of peripheral and central arterial catheters, wound care and bed changes, biochemical analysis, and intra-ICU procedures (activities 4,7,10,11,13,17). This suggests an equivalence in the responsibilities and tasks between the staff of the ICUs compared.

The second trend highlights that nursing assistants in ICU2 (Auxiliaries) perform the same functions as nurses in ICU1(Nurses), namely, ventilatory support, medication administration, drainage management, secretion suctioning, artificial airway management, nutrition, and procedures outside the ICU (activities 2,3,5,6,15,16,19). This finding indicates a redistribution of roles where nursing assistants in ICU2 (Auxiliaries) assume responsibilities that would normally be reserved for nurses in ICU1 (Nurses).

In the third trend, the functions of biochemical analysis, nutrition, medication administration, management of metabolic complications and central venous catheters, fluid balance, vasoactive agents, and forced diuresis are restricted for nursing assistants in the ICU-Nurses unit and are performed by registered nurses (activities 3,8,10,11,16). This restriction is in place to ensure that nursing assistants do not perform functions outside their technical competence and legal responsibility.

Regarding the average length of care required according to the patients' sociodemographic and clinical characteristics (Table 4), significant differences were found indicating that patients who underwent surgery, used vasoactive drugs prior to ICU admission, received intra-ICU blood transfusions, experienced adverse events, or died, required longer care times in both ICUs. Differences in care times by TISS-28 severity level were also significant in both ICUs (p < 0.001).

**Table 4:** TISS-28 care time required according to patient characteristics

Variables	ICU 1 Nurses		ICU 2 Assistants	
	Average ± SD	p-value	Average ± SD	p-value
Sex		0.877		0.919
Women	330.5 ± 101.63		309.53 ± 97.93	
Man	330.15 ± 88.41		309.96 ± 90.65	
Age range in years*		0.294		0.056
<40	323.03 ± 85.73		308.82 ± 92.33	
40–59	325.8 ± 94.52		295.46 ± 95.41	
60–79	341.07 ± 96.24		315.27 ± 93.68	
≥80	331.33 ± 115.31		348.52 ± 83.49	
Cardiovascular diagnosis		0.143		0.486
No	327.35 ± 92.67		311.85 ± 92.09	
Yes	340.9 ± 100.01		304.86 ± 97.72	
Digestive diagnosis		0.02		0.365
No	326.66 ± 94.44		308.19 ± 93.86	
Yes	358.45 ± 89.83		322.45 ± 92.68	
Neurological diagnosis		0.521		0.034
No	328.09 ± 96.63		303.38 ± 97.78	
Yes	333.71 ± 90.94		321.73 ± 84.67	
Surgical intervention*		<0.001		0.004
Non-surgical	307.88 ± 84.71		301.87 ± 97.61	
Programmed	319.48 ± 95.12		303.13 ± 63.33	
Urgent	374.71 ± 85.14		337.98 ± 95.85	
Vasoactive drugs prior to ICU admission		<0.001		<0.001
No	308.34 ± 89.14		293.31 ± 92.44	
Yes	363.34 ± 92.6		342.38 ± 87.83	
Category TISS 28*		<0.001		<0.001
II	155.29 ± 26.72		154.89 ± 28.83	
III	312.85 ± 54.9		306.97 ± 55.73	
IV	451.56 ± 35.28		446.14 ± 28.37	

ICU – Intensive Care Unit, SD – Standard Deviation, TISS 28 – Simplified Therapeutic Intervention Score System, * The Kruskal-Wallis test was applied to these variables, and the Mann-Whitney U test was applied to the other variables.

Another element identified in the results is the direct relationship between workload and negative patient health outcomes. For the two ICUs compared, the proportion of deaths, adverse events (AEs), and hospital stays longer than 7 days increased in direct proportion to the patients' TISS-28 category, meaning that patients with greater care requirements had worse health outcomes (Table 5). The ICU- 2 (Auxiliary) showed higher proportions of deaths, AEs, and prolonged stays at TISS-28 levels III and IV compared to the ICU-1 (Nurses), suggesting that, despite the overload present in both ICUs, the ICU-1 (Nurses) model provides safer care (Table 5). The proportion of deaths by TISS-28 level and the care times associated with this variable (Table 4) demonstrate the importance of this instrument as a predictor of mortality.

**Table 5:** Comparison of clinical outcomes by TISS-28 category

Variable – TISS Levels 28	ICU 1 Nurses			ICU 2 Assistants		
	% (Yes)	% (No)	p-value*	% (Yes)	% (No)	p-value*
Death			<0.001			<0.001
II	0 (0)	100 (51)		1.61 (1)	98.38 (61)	
III	7.14 (26)	92.85 (338)		11.56 (37)	88.43 (283)	
IV	33.33 (42)	66.66 (84)		49.35 (38)	50.64 (39)	
Adverse events			<0.001			<0.001
II	7.84 (4)	92.15 (47)		24.19 (15)	75.80 (47)	
III	47.80 (174)	52.19 (190)		59.06 (189)	40.93 (131)	
IV	80.16 (101)	19.84 (25)		92.20 (71)	7.79 (6)	
Stay >7 days			<0.001			<0.001
II	5.88 (3)	94.11 (48)		3.22 (2)	96.77 (60)	
III	24.45 (89)	75.54 (275)		30.62 (98)	69.37 (222)	
IV	46.83 (59)	53.17 (67)		67.53 (52)	32.46 (25)	

ICU - Intensive Care Unit, TISS 28 - Simplified Therapeutic Intervention Score System, * p value for chi square.

## Discussion

The analysis of the results will be presented in three levels or layers of analysis. First, the explicit findings indicated by the data regarding workload, patient characteristics, and nurse characteristics will be discussed. The second layer of analysis allows for an exploration of implicit aspects related to the organizational structures of nursing work in the ICU. The third layer proposes a broader level of considerations that reflect the value of care as a public good, including public policies and the generation of nursing knowledge. Finally, the limitations of the study will be assessed within the framework of the analyzed results.

Insufficient nurse allocation in the face of high patient dependency is the main factor generating work overload in the ICU, a common aspect in this study and in the literature consulted in Colombia and Latin America^5^,^11^,^13^,^20^,^24^-^26^. The studies show that, although most patients were classified as TISS-28 levels III and IV, the number of nurses assigned was always below that suggested by the instrument, with heterogeneous ranges from 1:3 nurses per patient to 1:12. It is worth noting that none of the studies mentioned indicate that patient allocation within a nursing team is based on the TISS-28 or any scale based on patient status; that is, the number of patients in the ward is divided among the number of nurses regardless of care requirements, generally by geography. Therefore, the TISS-28 would not be used as a criterion for distributing human resources according to patient severity.

A second point to highlight is that there are few studies that report the distribution of nursing assistants per patient11 and that analyze the skill The mix in relation to the TISS-28. In our study, the allocation of nursing assistants to ICU1-Nurses and ICU2-Assistants is appropriate compared to that estimated by the TISS-28 and coincides with that reported by Arango^11^. The shortage of nurses compared to the sufficiency of nursing assistants reveals how hospital institutions meet the need for human resources by hiring nursing assistants, because of regulatory gaps regarding the nurse- to-patient ratio in Colombia. In this regard, it should be noted that the regulations on minimum operating requirements for hospitals in force in Colombia in 1997^24^ established the obligation to assign 1 nurse for every 3 beds and one nursing assistant for every 2, but in subsequent reforms it was only established that the ICU must have a nurse and a nursing assistant available without considering the number of beds in operation.

The analysis of activity times in the TISS-28 for nurses and nursing assistants in ICU1-Nurses and ICU2- Assistants reveals, as do other studies, that most of the care time is dedicated to basic monitoring, cardiovascular and ventilatory support, and medication administration^26^. The distribution of functions between nursing assistants and nurses in ICU1-Nurses is consistent with their levels of training and legal responsibility, while in ICU2-Assistants, nursing assistants assume functions for which they lack the necessary level of competence and legal responsibility, such as the administration of inotropic and vasoactive medications, invasive airway management, and management of metabolic complications, to name just a few. The intra-ICU comparison shows that nursing assistants in ICU2-Assistants must assume a large part of the functions performed exclusively by nurses in ICU1-Nurses.

This phenomenon had already been mentioned by Arango^11^ and Ortega and Jiménez^15^ , who point out that the role of nurses is concentrated on administrative activities and some limited care, while nursing assistants are delegated direct care, which includes activities that, due to their level of knowledge and specialized techniques, are considered non-delegable. Blay and Roche^27^ agree on the same aspects and add the limited supervision of nurses over the work of nursing assistants. An important factor to consider is that neither nurses nor nursing assistants have influence over their work environments; that is, they are subject to the logic of delegation imposed by the institutional ways of organizing nursing work^17^.

The discussion presented leads to questioning to what extent the skill The mix of staff, skill level, and work environments influence patient safety and health outcomes. Data showed that the number of deaths, adverse events, and length of stay increased directly with the TISS-28 level, meaning that the increase in these negative outcomes can be explained by workload. Comparing the two ICUs, the proportion of negative outcomes was lower in the ICU with a higher percentage of nurses in the skill set. mix, suggesting that their level of qualification allows them to provide safer and higher quality care. These findings are consistent with studies such as those by Caballero, who reported a high and inverse correlation between workload and patient safety, presenting a correlation coefficient RHO = -0.658 and p<0.001^28^; Cho et al.^6^ who showed the effects of low staffing, as nurses responsible for the care of 8 or more patients rated patient safety and quality of care as poor or failing: (OR 5.97, CI 1.33– 26.8, p 0.001) and (OR 5.82, CI 2.91–11.7, p 0.001). Along the same lines, Alrabae et al.^29^ established negative correlations between workload and the perception of patient safety culture (r: -0.721 p < 0.001). Similarly, Aiken et al.^30^ demonstrated that overload and the low proportion of professional nurses in the skill These are factors related to the increase in in-hospital mortality.

The characteristics of patients associated with higher care requirements reported in similar studies are being male, admission for surgical pathology, experiencing adverse events, death, and hospitalization longer than 7 days^31^-^34^,^26^. These characteristics can guide preventive actions and highlight the importance of the instrument as a predictor of mortality and as a tool for managing the allocation and distribution of nursing staff, if its institutional use is adopted.

Regarding the second layer of analysis, it should be considered that one seemingly obvious consequence of work overload is that nurses are unable to fulfill their assigned tasks and omit them either partially or completely. From a quantitative perspective, Soliman^35^ established direct correlations between workload and omitted care (r = 0.730, p = 0.001). Omitted care was an emerging category in previous research conducted in the same ICUs, where care models were compared from a qualitative perspective^17^. Staff referred to the workload overload that led them to prioritize critically ill patients over less critically ill ones, biological interventions over emotional and family interventions, and to perform tasks less frequently, more quickly, and with lower quality^17^. A complementary perspective on this phenomenon is offered by Mesa and Romero, who point to the precarious working conditions in which nursing staff carry out their work, characterized by low wages, unstable employment relationships, and the "jack-of -all-trades" role imposed upon them to attend to the multiplicity of functions (many of them unrelated to care), which leads them to perform "care in a rush" or against the clock, which has negative implications for quality^36^. These types of work environments encourage care to be focused on functions and not on people^17^.

Regarding the third layer of analysis, it is worth asking: What other realities are hidden behind the presented data? And what do they suggest in terms of valuing care as a public good? Based on these questions, this paper aims to analyze some contradictions revealed by the data considering certain elements of the social organization of care, which together demonstrate an undervaluation of nursing care as a public good. Menéndez Spina^37^ highlights the importance of studying the health-illness- care-attention processes as "spies" that reveal social contradictions and underscores the contribution of these contradictions to problematizing reality, offering keys to interpreting the dominant social order. The contradictions regarding care are evident when considering that nursing staff constitute more than 50% of the healthcare workforce, and that in 2018 a campaign called Nursing was carried out. Now, to highlight and strengthen women's leadership in the health sector, which during the Covid-19 pandemic saw nurses praised in the media as heroines risking their lives to care for those affected. However, this contrasts sharply with the fact that in 2024, nursing staff continue to perform their work with a 39% gender pay gap compared to the medical profession^38^, without recognition of their postgraduate training, under conditions of physical and mental overload, and their work is being replaced by nursing assistants, who are cheaper for the system but whose work undermines the quality of care and puts patients' health at risk^15^.

At the policy level, it is evident that the regulations of the Colombian health system have not established a nurse-to-patient ratio; care is conceived as a hotel expense within hospital fee schedules^39^; and, although there is a National Nursing Policy and a ten-year plan for 2020-2030 that was developed with the participation of nurses from all over the country, there are few results in terms of transforming working conditions and generating knowledge to address adverse working conditions and the contributions of care to patient safety and quality of care. In other words, a policy instrument exists, but the necessary agency as a political subject that would allow the profession to advance in transforming its current conditions has not been developed.

Taken together, the arguments presented regarding the microsocial space of workload and the macro dimension where economic and political forces shape the contexts of care delivery allow us to affirm that a process of devaluation of nursing care as a public good is underway in Colombian society, despite its central importance in sustaining and maintaining life. It is primarily the responsibility of the nursing profession to organize, work, and mobilize to problematize this situation, raise awareness of it, and seek alternatives for transformation.

Limitations of the study include the implementation timeframe for the pilot study of the two nursing care models compared (2018-2020), which restricted the data collection period. Furthermore, research development timelines are limited, especially when part of the research team consists of nurses working in adult intensive care units, meaning that the knowledge products derived from the study are generated after the research projects have been concluded.

## Conclusions

It was identified that both ICUs have a higher workload for nurses, exceeding the ideal number of patients proposed by TISS-28, and that their duties are incompatible with the time available per patient. Some care provided by nursing assistants in ICU-2 does not correspond to their level of training and legal responsibility, such as administration of medications and vasoactive drugs, ventilatory and nutritional support, and artificial airway management. Clinical characteristics of the patients, such as vasoactive drug use prior to ICU admission, transfusions, adverse events, and death, were correlated with longer care times.

A direct correlation was found between the incidence of adverse events (AEs) , the proportion of deaths, hospital stays longer than 7 days, and the TISS-28 score in both ICUs, indicating that the nurses in ICU-1 achieved better results in terms of safety and quality of care. TISS-28 has proven to be a useful instrument for measuring workload; however, it does not reflect some of the activities performed by nurses, especially administrative, educational, and socio-emotional tasks. Therefore, future research is recommended to adapt or create instruments that reflect the complexity of nursing work in the ICU.
